# Comparison of Exoelectrogenic Bacteria Detected Using Two Different Methods: U-tube Microbial Fuel Cell and Plating Method

**DOI:** 10.1264/jsme2.ME11205

**Published:** 2011-12-01

**Authors:** Jaecheul Yu, Sunja Cho, Sunah Kim, Haein Cho, Taeho Lee

**Affiliations:** 1 Department of Civil and Environmental Engineering, Pusan National University, Busan 609–735, Korea

**Keywords:** electricity, exoelectrogen, dilution to extinction, U-tube microbial fuel cell

## Abstract

In a microbial fuel cell (MFC), exoelectrogens, which transfer electrons to the electrode, have been regarded as a key factor for electricity generation. In this study, U-tube MFC and plating methods were used to isolate exoelectrogens from the anode of an MFC. Disparate microorganisms were identified depending on isolation methods, despite the use of an identical source. Denaturing gel gradient electrophoresis (DGGE) analysis showed that certain microorganisms became dominant in the U-tube MFC. The predominant bacterium was similar to *Ochrobactrum* sp., belonging to the *Alphaproteobacteria*, which was shown to be able to function as an exoelectrogen in a previous study. Three isolates, one affiliated with *Bacillus* sp. and two with *Paenibacillus* sp., were identified using the plating method, which belonged to the Gram-positive bacteria, the *Firmicutes*. The U-tube MFCs were inoculated with the three isolates using the plating method, operated in the batch mode and the current was monitored. All of the U-tube MFCs inoculated with each isolate after isolation from plates produced lower current (peak current density: 3.6–16.3 mA/m^2^) than those in U-tube MFCs with mixed culture (48.3–62.6 mA/m^2^). Although the isolates produced low currents, various bacterial groups were found to be involved in current production.

Microbial fuel cells (MFCs) have been considered to be a promising technology in the field of sustainable and renewable energy. An MFC is a system for generating electricity from organic compounds using microorganisms as a bio-catalyst. In the anode chamber of MFCs, microorganisms degrade organic compounds, such as glucose, acetate and ethanol, etc., and the electrons produced from this degradation are transferred to the anode as an electron acceptor. The microorganisms capable of transferring electrons to the anode are called “exoelectrogens”. Exoelectrogens function as electrochemically active bacteria, capable of transferring electrons from their cell body to outside of the cell, and play an important role in electricity generation (7, 8). In general, there are direct and indirect ways for exoelectrogens to transfer electrons. Microorganisms transfer electrons directly by developing a biofilm on the anode surface or indirectly through electron shuttles that exist in the anodic suspension. Current can be generated from differences in the potential due to the movement of electrons; they contribute to the production of electricity in both ways; however, such information on electron transfer mechanisms is still insufficient to understand the physiology of the exoelectrogen, the ecology of anodic microbial communities on the electrodes, and the relationship between the exoelectrogen and other bacteria. Therefore, the identification and characterization of exoelectrogens are the most significant factors for increasing the efficiency of transfer electrons and producing higher power via MFCs (7, 8).

Methods for the isolation of exoelectrogens from the anode of MFCs can be categorized as follows: dilution to extinction and plating methods. The plating method is known as a generally convenient method to isolate exoelectrogens from MFC anodes. So far, a great number of exoelectrogens isolated from MFC anodes have been reported. There have also been many investigations on these bacteria, such as *Clostridium butyricum*(10), *Aeromonas hydrophila*(13), *Rhodoseudomonas palustris*(17), *Aeromonas* sp. (2) and *Acrobacter butzleri*(3). The main advantage of the plating method for their isolation is the conventional and relatively convenient experimental process. With plating, however, it is possible that it will discover not only exoelectrogens but also other bacteria that are not able to transfer electrons extracellularly on an MFC anode.

The exoelectrogens identified by the plating method are also known as dissimilatory Fe (III) reducing bacteria, which are able to reduce insoluble iron (7). One reason is that the media for metal-reducing bacteria have been used to isolate exoelectrogens, even though not all of the microorganisms growing in the media are exoelectrogens. Therefore, microorganisms were generally identified by their electrochemical activity after isolation (by plating). It was impracticable to observe the electrochemically activity of bacteria discovered in the microbial community in MFCs. Moreover, the cultivation-dependent method, the plating method, is well known for significant limiting the numbers and populations of bacteria that represent the entire microbial community.

The dilution to extinction method, a different and cultivation-independent method for the isolation of exoelectrogens devised in previous studies, is an alternative method that enables exoelectrogens to be isolated by continuous monitoring of the electricity produced in MFCs. With this method, dominant strains of anode respiring bacteria or the electrochemically active microbial community can be isolated. *Ochrobactrum anthropi*(20) and *Comamonas denitrificans*(16) have been reported as microorganisms isolated via the dilution to extinction method. *O. anthrophi* YZ-1 isolated from U-tube MFCs using the dilution to extinction method showed lower maximum current density of 708 mA/m^2^, but higher coulombic efficiency of 93% than the mixed culture (1730 mA/m^2^). They produced current using a wide range of substrates (acetate, lactate, propionate, butyrate, glucose, sucrose, cellobiose, glycerol, and ethanol) (20).

Most of the defined strains isolated by the plating method have revealed the capability of reducing metals. It is unclear whether electrochemically active metal-reducing bacteria can significantly affect electricity generation; thus it should be considered that metal-reducing bacteria are really major exoelectrogens in MFC. However, previous studies have generally been carried out using a single method, or with different studies using different inocula, which have made it difficult to collectively compare studies. Thus, in this study, we used two methods, the dilution to extinction and plating methods, to identify and to isolate exoelectrogen. Through dilution to extinction experiments using a U-tube MFC, we tried to reduce the diversity of the exoelectrogenic community and to identify exoelectrogen. Then we isolated iron-reducing bacteria from the anodic microbial community of U-tube MFC by the plating method, and they were compared to the exoelectrogen identified using U-tube MFC.

## Materials and Methods

### U-tube MFC construction

A U-tube MFC was constructed so that exoelectrogens could be easily enriched and settled on the anode. The anode chamber (10 mL) in the MFC was a cylindrical rod-shape and the cathode chamber (30 mL) an asymmetrical U-shape (20). Sodium acetate (1 g/L) was used as the electron donor, and the composition of anode medium was as follows (per liter): NH_4_Cl 0.23 g, MgSO_4_·7H2O 0.2 g, K_2_HPO_4_ 6.8 g, KH_2_PO_4_ 8.8 g, NaCl 0.5 g, and 1 mL of a trace metal solution. In the cathode chamber, 100 mM ferrocyanide, with 100 mM PBS, was used as the catholyte. All solutions were autoclaved before injection and the pH was typically controlled to 7. The anode and cathode chambers were separated by a proton exchange membrane (Nafion 117; DuPont, USA). Grphite felt and carbon cloth were used as the anode and cathode, respectively. Titanium wire was set to function as the current collector. The circuit between the anode and cathode was connected by a 1000 Ω external resistor. N_2_ gas was purged after injecting solutions into each chamber.

### Isolation using U-tube MFC

**Fig. 1:**
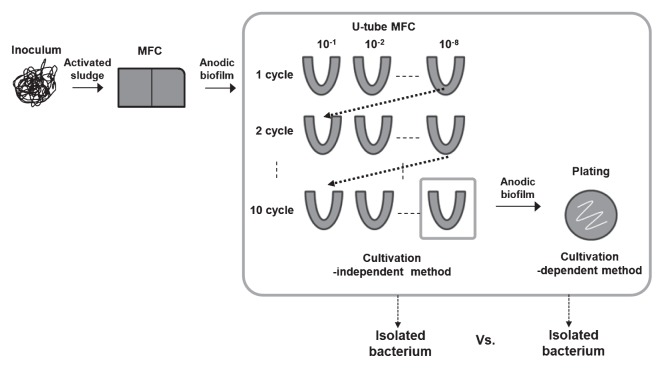
Experimental flow chart to compare exoelectrogens detected by two different methods.

The initial inoculum was a cell suspension separated from a piece of the anode of a lab-scale two-chamber MFC fed with acetate for 1 year. The excised anode from the MFC was transferred to a test tube, which also contained glass beads and 10 mL of 50 mM phosphate-buffered saline (PBS), using the tube, and then mixed by vortexing to obtain the cell suspension from the anode electrode (1 cm^2^). The cell suspension in the tube was serially diluted 10-fold in steps to 10^−8^. The diluted suspensions were added to 8 U-tube MFCs, which were operated with 3 times in batch mode as the 1^st^ cycle. Then, the anode cell of U-tube MFC inoculated with the dilution suspension of 10^−8^ was serially diluted from 10^−1^ to 10^−8^ again. U-tube MFCs were operated in the same way (Fig. 1). The experimental process was repeated 10 times. A U-tube MFC, with only sterile medium and without an inoculum in the anode chamber, was used as the control. U-tube MFCs were operated in the same way as in Zuo *et al.*(20) at a constant temperature of 30°C. The medium in the U-tube MFC was refreshed when the voltage decreased below 10 mV. The medium in the reactor showing the minimum current for that dilution among all the U-tube MFC reactors was used in the next dilution step.

### Isolation using plating method

The inoculum used in plating isolation was obtained from the anode cell suspension of the 10th cycle of the U-tube MFCs. The cell suspension was diluted to 10^−1^ and 10^−3^, and the diluted solutions spread onto agar plates. Sodium acetate (1 g/L) was used as the electron donor, and the composition of the medium used for the isolation of Fe (III)-reducing bacteria was as follows (per liter of distilled water): NaHCO_3_ 2.5 g, CaCl_2_·2H_2_O 0.1 g, KCl 0.1 g, NH_4_Cl 1.5 g, NaH_2_PO_4_ 0.6 g, NaCl 0.1 g, MgCl_2_·6H_2_O 0.1 g, MgSO_4_·7H_2_O 0.1 g, MnCl_2_·4H_2_O 0.005 g, NaMoO4·2H_2_O 0.001 g, and yeast extract 0.05 g. Amorphous ferric oxyhydroxide was added to provide ca. 250 mmol Fe (III) per liter. Samples were cultured in an anaerobic chamber at 30°C. After 25 days, the dominant colonies were classified morphologically and then streaked onto other agar plates. The cultured isolates were transferred to 1.5 mL tubes and stored with 40% glycerol at −80°C.

### Electrical analysis

The voltage output was measured using a data acquisition system (Model 7700; Keithley Instruments, OH, USA) every 2 min. The voltage, current density and power density were calculated using the following equations: V = IR, Cd = I/A, Pd = VI/A, where V is the voltage; I, current; R, external resistance; Cd, current density; A, anode surface and Pd, power density. Current density and power density were normalized to the anode’s projected surface area.

### 16S rRNA gene analysis

After each dilution step had been completed, genomic DNA from the anode of the most diluted U-tube MFC generating electricity was extracted and partial 16S rRNA genes (V1-3 region) were amplified as described by Yu *et al.*(18). The DNA template (80 μg) was added to the PCR solution. The detailed PCR primer and condition are described in Table 1. For the denaturing gel gradient electrophoresis (DGGE) analysis, denaturing acrylamide gel (6% of acriamide-bisacrylamide 37.5:1) was prepared with a urea gradient from 14.7 to 25.2% containing 14% formamide. Samples were loaded onto the denaturing acrylamide gel and electrophoresis was performed at 20 V for 15 min followed by 200 V for 360 min in 0.5×TAE (40 mM Tris base with 1.0 mM EDTA and 20 mM sodium acetate at pH 7.4) at a constant temperature of 60°C using a D-Code system (Bio-Rad Laboratories, Hercules, CA, USA). After electrophoresis, the DGGE was visualized and several bands were extracted as described by Yu *et al.*(18). In the plating method used for isolating exoelectrogens, colony PCR of all isolates was performed using EUB 27F and EUB 518R primers. PCR products were subjected to electrophoresis and extracted using a gel extraction kit (Solgent, Daejeon, Korea). The extracted samples were sequenced as described by Yu *et al.*(18). The 16S rRNA gene sequences were searched for the most closely related strains using the BLAST program within the NCBI database.

### Nucleotide sequence accession numbers

The partial 16S rRNA gene sequences of three colonies shown to be different via the plate method in this study were deposited in the GenBank database under accession numbers JM561791–JM561793. The partial 16S rRNA gene sequences obtained from DGGE were also deposited in the GenBank database under accession numbers HM161758–HM161761.

## Results

### Isolation using serially diluted U-tube MFCs

**Fig. 2:**
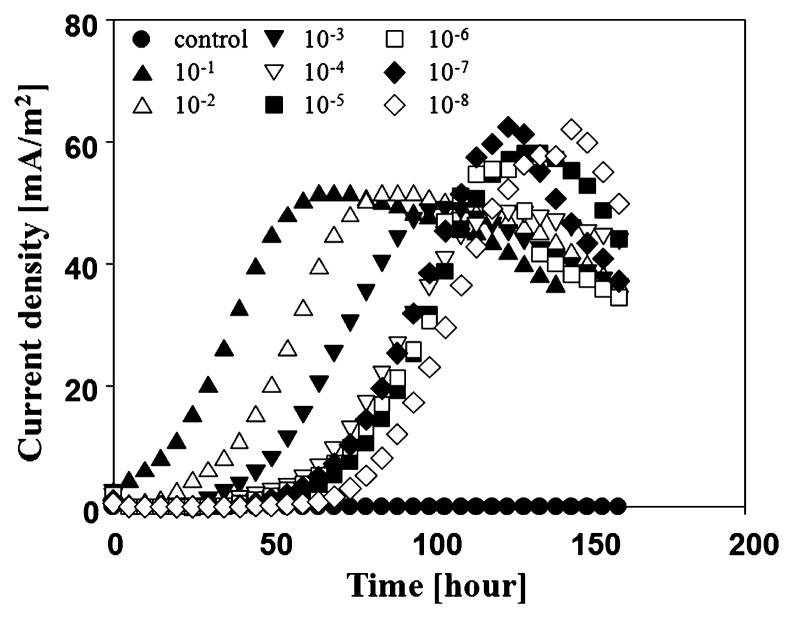
Current density trend of 10^th^ cycle using U-tube MFC.

The re-suspended solution of an anode electrode was serially diluted (10^−1^–10^−8^) and the diluted cell suspension was injected into each anode chamber of 8 U-tube MFCs fed with acetate. The current in each MFC after dilution was monitored continuously until it had decreased below 10 mV. All U-tube MFCs (10^−1^–10^−8^) showed peak current densities of about 48–62 mA/m^2^. The duration of the lag phase increased from 20 h for the lowest dilution (1^st^ dilution at 10^th^ cycle) to 60 h for the highest dilution (8^th^ dilution at 10^th^ cycle) (Fig. 2). The abiotic U-tube MFC (control) produced no current.

### Anodic microbial community analysis

**Fig. 3:**
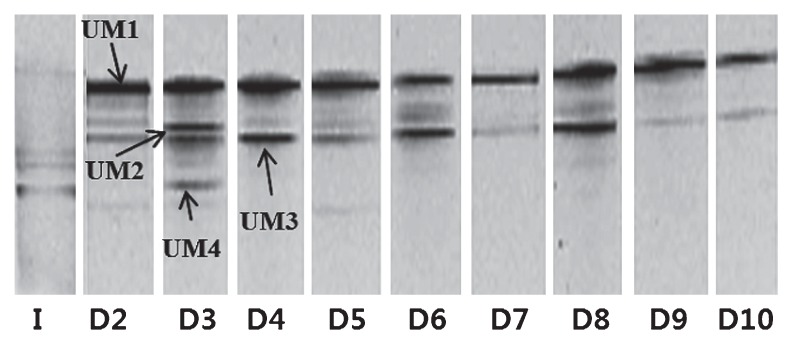
DGGE band profile of 16S rRNA fragments amplified for anodic biofilm of U-tube MFC after dilution to 10^−8^ from 2^nd^ to 10^th^ cycle; I: inoculation sample, D2: 10^−8^ dilution at 2nd cycle, D3: 10^−8^ dilution at 3rd cycle, D4: 10^−8^ dilution at 4th cycle, D5: 10^−8^ dilution at 5th cycle, D6: 10^−8^ dilution at 6th cycle, D7: 10^−8^ dilution at 7th cycle, D8: 10^−8^ dilution at 8th cycle, D9: 10^−8^ dilution at 9th cycle and D10: 10^−8^ dilution at 10th cycle.

Several bands showed higher intensities on the DGGE gel with respect to the dilution steps (Fig. 3). The microorganisms were assumed to represent bands of higher intensity and therefore would have the potential to transfer electrons and generate electricity in a U-tube MFC. Band UM1 was dominant in the U-tube MFC and was affiliated with *Ochrobactrum* sp., which has been reported as an exoelectrogen (Table 2). This exoelectrogen has not previously been reported as an Fe (III)-reducing bacterium. Bands UM2 and UM4 vanished during the early dilution step in serial U-tube MFCs. Band UM3 disappeared after 8 cycles. Band UM 2 was related to *Achromobacter* sp., which has been detected in an MFC. Zhu *et al.*(19) reported that *Achromobacter* sp. was isolated in enrichment culture tolerating and reducing Cr (VI) at extremely high concentrations. The closest bacterium to band UM 3 also belonged to *Ochrobactrum* sp. Band UM 4 was closely related to *Acinetobacter* sp., which has been reported as a community member in previous MFC studies (1, 5).

### Isolated microorganism using plating method

The anode cell suspension of the 10^th^ cycle was diluted to 10^−1^ and 10^−3^, and the diluted solutions spread onto agar plates. The plates were cultured in an anaerobic chamber at 30°C. After 25 days, the dominant colonies were classified morphologically and then streaked onto other agar plates. Among the colonies on the plates, three (PM 1, PM 2 and PM 3) were differentially selected due to their morphologies, with all identified as Gram-positive bacteria from their 16S rRNA sequence analysis. So far, there have been a few MFC studies in which Gram-positive bacteria were reported as exoelectrogens; Rabaey *et al.*(14, 15) reported that *Enterococcus* spp., Gram-positive bacteria, were detected in an MFC and *Brevibacillus* spp. were dominant in the anodic microbial community of the MFC (12). In this study, PM 1, 2 and 3 were identified as *Bacillus sonorensis*, *Paenibacillus pabuli* and *P. amylolyticus*, respectively (Table 3).

### Current production by isolates

**Fig. 4:**
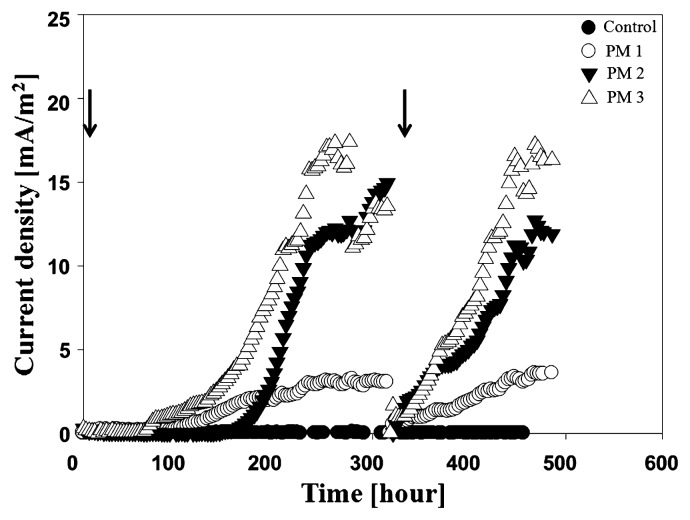
Current density from U-tube inoculated with PM1, PM2 and PM3 isolated using the plating method; the arrows indicated substrate feeding.

A current production test was conducted to confirm the electrochemically activities of PM1, 2 and 3. The culture solution of the PMs (−3×10^8^ cells/mL) was inoculated into the U-tube MFCs, which were operated in batch mode. All the isolates produced relatively lower peak current densities of 3.6–16.3 mA/m^2^ than the U-tube MFC inoculated with mixed cultures (Fig. 4). Of the isolates, PM 3 exhibited the highest peak current density, 16.3 mA/m^2^. Rabaey *et al.*(14, 15) reported that *Enterococcus* spp., Gram-positive bacteria, generated a very low current in a pure culture with acetate as the electron donor.

## Discussion

We used two different methods, the U-tube MFC and plating method, to compare exoelectrogen isolated from identical inoculum U-tubes. To identify exoelectrogens that produced a current, a cultivation-independent technology, PCR-DGGE, was used, and then DNA band fragments on the DGGE-gel were sequenced; however, we could not isolate exoelectrogens using U-tube MFC and 3 species, *Ochrobactrum* sp., *Achromobacter* sp. and *Acinetobacter* sp. were observed in U-tube MFCs. Among them, the most dominant bacterium was closely related to *Ochrobactrum* sp. belonging to the *Alphaproteobacteria. Achromobacter* sp. is a Gram-negative genus bacterium belonging to the *Betaproteobacteria* and can use a variety of organic acids and aminoacids as carbon sources. *Acinetobacter* sp. is a Gram-negative bacterium included in the *Gammaproteobacteria* and is an important soil bacterium (4).

Interestingly, the isolates obtained via the plating method were affiliated with *Bacillus* sp. and *Paenibacillus* sp., belonging to the *Firmicutes. Bacillus* spp. and *Paenibacillus* spp. are groups of Gram-positive, spore-forming, and rod-shaped bacteria. In particular, *Paenibacillus* spp. were separated from *Bacillus* spp., which are a genus of Gram-positive, facultative anaerobic, endospore-forming bacteria and important members of soil or plant-associated ecosystems (6). Petrie *et al.*(11) reported that *Paenibacillus* spp. were predominant in iron-reducing consortia in contaminated sediments. In a previous study, it was reported that *Bacillus* sp. can generate current in MFCs (9); however, it has not been reported whether *Paenibacillus* sp. has the ability to function as exoelectrogens in MFCs. All U-tube MFCs inoculated with each isolate after isolation from plates produced a lower current (peak current density: 3.6–16.3 mA/m^2^) than those in U-tube MFCs with mixed culture (48.3–62.6 mA/m^2^). This shows that the exoelectrogenic activity of a complex microbial community is more effective than that of pure isolates in MFCs. Some MFC studies have reported that the power generated from pure cultures was much lower than from mixed cultures (12, 14, 20). If some strains directly transfer electrons to the electrode, current could be immediately generated in MFC when substrate was added to MFC. In this study, however, U-tube MFC inoculated with the isolated strain showed a long lag phase before current generation, which indicated that the strain might produce electron mediators for the transfer of electrons to the electrode. Some *Paenibacillus* sp., such as *P. polymyxa*, produce antibiotics, which could be used as exogenous mediators, as is the case with phenazines produced by *Pseudomonas aeruginosa*(6, 15). In general, *Bacillus* sp. and *Paenibacillus* sp. use glucose as a carbon source. In this study, however, acetate was used as the electron donor in MFC. Thus, U-tube MFC might produce a low current because acetate is not an appropriate carbon source.

**Fig. 5:**
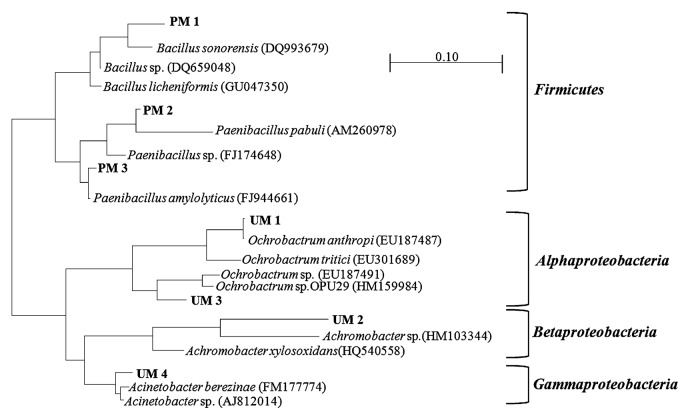
Phylogentic tree of isolates obtained in this study and closely related species based on 16S rRNA gene sequences. The tree was constructed with the neighbor-joining method in Arb.

The phylogenetic tree showed that the isolated/identified exoelectrogens can differ significantly, although an identical inoculum source was used (Fig. 5). Isolates from the conventional plating method were included in the *Firmicutes*, while those identified by DGGE from U-tube MFCs belonged to proteobacterial groups.

This is the first study to show that *Paenibacillus* sp. has the ability to generate electricity, which indicates that many bacteria might be related to those that produce electricity in MFCs; however, additional study is required to elucidate further information about the metabolism, the electron transfer mechanism within *Paenibacillus* sp. and their role in the anodic microbial community.

## Figures and Tables

**Table 1:** PCR primers and conditions used in this study

Primer	Sequence (5′ to 3′)	PCR conditions	Target
Eub 27F(GC)	AGT TTG ATC CTG GCT CAG	2 min 95°C, followed 30 cycles of 20 s at 95°C, 40 s 55°C, 30 s 72°C followed by a 3 min final extension at 72°C	Bacteria
Eub 518R	ATT ACC GCG GCT GCT GG		V 1, 2 and 3 region

F: forward primer, R: reverse primer, GC: GC clamp (CGC CCG CCG CGC GCG GCG GGC GGG GCG GGG GCA CGG GGG G) was attached to the 5′ end for DGGE

**Table 2:** 16S rRNA gene sequences of DGGE band

Band name	Phylum	Closest 16S rRNA sequence	Accession No.	Similarity (%)
UM 1	*Alphaproteobacteria*	*Ochrobactrum anthropi*	EU187487	100
		*Ochrobactrum* sp. OTU29	HM159984	100
		*Ochrobactrum tritici*	EU301689	100

UM 2	*Betaproteobacteria*	*Achromobacter* sp.	HM103344	100
		*Achromobacter* sp. QXH	JN043371	99
		*Achromobacter xylosoxidans*	HQ540558	99

UM 3	*Alphaproteobacteria*	*Ochrobactrum* sp.	EU187491	97
		*Ochrobactrum* sp. OTU29	HM159984	96
		*Uncultured alphaproteobacteria bacterium*	CU918533	96

UM 4	*Gammaproteobacteria*	*Acinetobacter* sp.	AJ812014	96
		*Uncultured Acinetobacter* sp.	EU705310	96
		*Acinetobacter berezinae*	FM177774	96

**Table 3:** Identification of isolates by a plating method using sequences of partial 16S rRNA genes

Name	Phylum	Closest 16S rRNA sequence	Accession No.	Similarity (%)
PM 1	*Firmicutes*	*Bacillus sonorensis*	DQ993679	100
PM 2	*Firmicutes*	*Paenibacillus pabuli*	AM260978	99
PM 3	*Firmicutes*	*Paenibacillus amylolyticus*	FJ944661	96
